# Enhancing Therapy Adherence: Impact on Clinical Outcomes, Healthcare Costs, and Patient Quality of Life

**DOI:** 10.3390/medicina61010153

**Published:** 2025-01-17

**Authors:** Urszula Religioni, Rocío Barrios-Rodríguez, Pilar Requena, Mariola Borowska, Janusz Ostrowski

**Affiliations:** 1 School of Public Health, Centre of Postgraduate Medical Education of Warsaw, 01-813 Warsaw, Poland; 2 Departamento de Medicina Preventiva y Salud Pública, Universidad de Granada, 18012 Granada, Spainprequena@ugr.es (P.R.); 3 Instituto de Investigación Biosanitaria de Granada (ibsGRANADA), 18016 Granada, Spain; 4 Centro de Investigación Biomédica en Red de Epidemiología y Salud Pública (CIBERESP), 28029 Madrid, Spain; 5 Department of Cancer Epidemiology and Primary Prevention, Maria Sklodowska-Curie National Research Institute of Oncology, 00-001 Warsaw, Poland

**Keywords:** adherence, healthcare costs, clinical efficacy, quality of life

## Abstract

Adherence to therapy, defined as the extent to which a patient follows prescribed therapeutic recommendations, is a pivotal factor in the effective management of chronic diseases such as diabetes, hypertension, and cardiovascular conditions. This review highlights the profound influence of adherence on clinical outcomes, healthcare costs, and patient quality of life. Despite its critical importance, non-adherence remains a pervasive challenge globally, contributing to suboptimal treatment results, higher rates of complications, increased hospitalizations, and substantial healthcare expenditures. This narrative review examines the multifaceted impact of adherence, focusing on its role in achieving clinical efficacy, mitigating economic burdens, and enhancing patient well-being. The findings reveal that poor adherence exacerbates the risk of disease progression, complications, and higher healthcare costs. Conversely, improved adherence promotes better disease control, fewer complications, and enhanced patient quality of life. Interventions such as patient education, streamlined treatment regimens, and the integration of digital health tools have shown promise in addressing adherence barriers. Furthermore, the role of healthcare professionals is underscored as fundamental, with their continuous support, effective communication, and efforts to build patient trust being essential to fostering better adherence. In conclusion, adherence significantly affects clinical outcomes, healthcare costs, and patient quality of life. Addressing barriers to adherence requires a comprehensive and personalized approach, considering individual patient needs and circumstances. Future research should prioritize the long-term evaluation of emerging technologies and the development of tailored strategies to improve adherence across diverse patient populations. Strengthening adherence is not only crucial for individual patient outcomes, but also for enhancing the sustainability and efficiency of healthcare systems.

## 1. Background

The World Health Organization defines adherence to long-term therapy to the extent to which a person’s behavior—taking medication, following a diet, and/or executing lifestyle changes—corresponds with agreed recommendations from a healthcare provider [1]. The terms “adherence” and “compliance” have often been used interchangeably, though subtle distinctions exist between the two of them. Compliance traditionally implied a passive role for the patient, who is expected to follow the healthcare provider’s instructions without deviation. In contrast, adherence emphasizes a more collaborative approach, where the patient actively participates in their treatment plan, aligning with the principles of patient-centered care [2].

Persistence, another related term, refers to the duration of time a patient continues the treatment as prescribed, without discontinuation [3]. Both adherence and persistence are necessary to achieve therapeutic goals, but they focus on different aspects of patient behavior. While adherence is often measured by the proportion of prescribed doses taken, persistence is concerned with the continuity of treatment over time.

Chronic diseases such as diabetes, hypertension, and cardiovascular diseases constitute a major public health problem due to their significant morbidity and mortality [4,5,6]. Despite the advancements in the treatment of these diseases over recent decades, a high level of adherence is required to achieve the optimal effects of therapy. In this context, adherence refers to the degree to which patients follow medical recommendations regarding both treatment regimens and lifestyle changes or regular check-ups [3,7]. Non-adherence to these recommendations continues to pose a significant challenge for healthcare systems globally [8]. This issue is particularly critical, as consistent, long-term adherence is essential to achieve optimal therapeutic outcomes and improve patients’ quality of life in the management of these diseases. The growing prevalence of these conditions further underscores the urgency of addressing this problem. Without effective interventions to enhance adherence, the burden of these diseases is likely to escalate, leading to higher healthcare costs, increased rates of hospitalizations, and preventable complications that compromise patient health and well-being [9,10,11].

Understanding the seriousness of this phenomenon is becoming essential for appropriate disease management at both the individual and population levels. The reasons behind the low rates of adherence are due to both patient and health system characteristics [12]. Therefore, the gap between the expected adherence and the actual patient behavior highlights the need for innovative strategies to address this problem. While various systematic reviews have explored the underlying causes of non-adherence to therapy, including complex treatment regimens, side effects, and socioeconomic barriers [8,13,14], a comprehensive understanding of both individual and population-level consequences may help to effectively target interventions.

Taking the above into account, this article provides a comprehensive review of the current state of knowledge on how adherence affects the effectiveness of chronic disease treatment, the economic consequences of non-adherence, and the impact on quality of life, and it identifies strategies that can be implemented to improve adherence in patients with chronic diseases.

## 2. Adherence and Challenges

Adherence is a complex issue of a multifaceted nature that is influenced by a range of individual and structural factors. This complexity makes the assessment a challenging process. To address this, direct and indirect methods are employed. Direct methods, such as biochemical assays to detect drug levels in bodily fluids, are considered more accurate but are invasive and costly. Indirect methods include patient self-reports, prescription refill records, and pill counts. While these methods are less invasive and easier to implement, they are prone to biases such as social desirability or recall inaccuracies [15,16]. More recently, the use of electronic monitoring systems and mobile health applications has emerged as a promising tool for real-time tracking of medication adherence, providing more reliable data while engaging patients in their own care [17]. An analysis of patient-reported outcome measures to assess adherence to treatment among patients with cardiovascular disease and/or type 2 diabetes showed that questionnaires assessing medication adherence among these groups of patients do not meet the criteria to be classified as trustable. Some of them (e.g., 8-item Morisky Medication Adherence Scale, Simplified Medication Adherence Questionnaire, Medication Adherence Questionnaire, or specific questionnaires, e.g., to evaluate the ART (Antiretroviral Therapy) adherence) have the potential to be recommended for use, but further research is needed to ensure their quality [18,19].

Studies show that medication non-adherence is a global problem, the scope and nature of which vary depending on the region and population. In high-income countries, such as the United States of America and Australia, the percentage of patients who do not adhere to the recommendations for chronic therapy ranges from 14% to 45% [8,20,21]. However, non-adherence rates are even higher in low- and middle-income countries. Although several studies in the United States have reported a downward trend in medication non-adherence, this trend has not been observed worldwide [22]. The exact reasons for regional differences are difficult to determine, but may include differences in culture and beliefs, the use of alternative medicine, the structure of the healthcare systems, and the affordability and availability of medications.

Importantly, observational studies indicate that adherence to medication recommendations shows gender differences and is on average lower in women than in men. Sociodemographic and psychological factors (younger age, non-white ethnicity, low educational level, low income, and depressive disorders), as well as the specific diseases and treatments, may explain this sex-related difference [23]. Additionally, compared to men, women are more in favor of lifestyle changes than pharmaceutical treatment [24].

**Figure 1:**
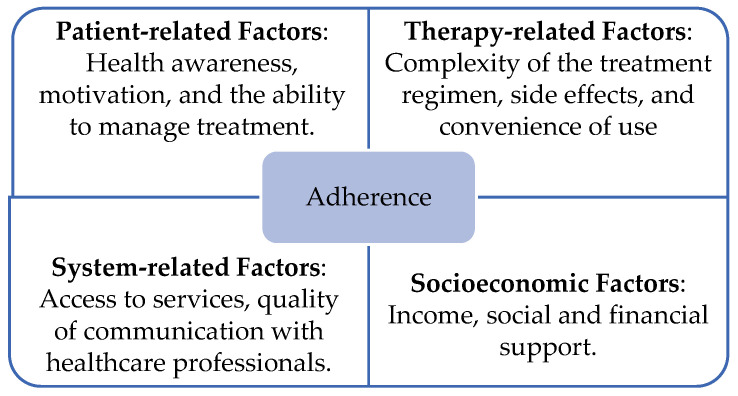
Key factors influencing adherence.

Multiple factors contribute to non-adherence, and understanding these different drivers is essential in designing targeted interventions (Figure 1), as follows:

1. Patient-Related Factors: Patients’ beliefs, knowledge, and psychological factors significantly influence adherence. For example, low health literacy and poor understanding of the disease and its treatment can lead to the incorrect use of medication [25]. Mental health conditions such as depression and anxiety are also associated with low adherence, particularly in patients with chronic illnesses [14,19].
2. Therapy-Related Factors: The complexity of treatment regimens, including the frequency and number of medications, plays a significant role in adherence. Polypharmacy, common among elderly patients, increases the risk of medication errors and reduces adherence [26]. Additionally, side effects, particularly in long-term treatments, can discourage patients from continuing therapy as prescribed.
3. Healthcare-System-Related Factors: Healthcare system barriers include limited access to care, inadequate patient–provider communication, and insufficient follow-up mechanisms. The quality of the patient–provider relationship is crucial, as poor communication and lack of trust can undermine adherence [27,28]. Moreover, structural barriers such as high out-of-pocket costs and limited access to medications in certain regions further exacerbate non-adherence.
4. Socioeconomic Factors: Socioeconomic status is one of the most significant predictors of adherence. Patients with lower income levels often struggle with the cost of medications, which can lead to rationing or skipping doses [10]. In addition, social support networks, or the lack thereof, can either encourage or hinder adherence behaviors.

Taking into consideration all of these factors, medication non-adherence might be categorized into intentional and unintentional. Intentional non-adherence occurs when a patient consciously decides not to follow the prescribed regimen due to factors such as perceived ineffectiveness, fear of side effects, or mistrust in the healthcare system. Unintentional non-adherence, on the other hand, results from forgetfulness, confusion about the treatment regimen, or financial barriers [25].

## 3. Adherence and Clinical Outcomes

In chronic diseases, adherence to therapy is fundamental to achieving optimal treatment outcomes. Specifically, diabetes management requires consistent adherence to medication, dietary guidelines, and physical activity regimens. Studies have shown that patients who adhere to prescribed antidiabetic medications experience better glycemic control, leading to reduced incidences of microvascular and macrovascular complications, such as retinopathy, nephropathy, and cardiovascular events [29]. In addition, adherence to insulin therapy, oral hypoglycemic agents, and lifestyle modifications has been shown to decrease the risk of severe complications, including amputations, blindness, and end-stage renal disease [30]. The long-term benefits of improved adherence also include a lower risk of cardiovascular events, such as myocardial infarction and stroke, which are common comorbidities in diabetic patients. Poor adherence, on the other hand, has been associated with higher levels of glycated hemoglobin (HbA1c), which indicates suboptimal blood sugar control and an elevated risk of diabetes-related complications [31].

Similarly, adherence to antihypertensive medications is essential for achieving and maintaining target blood pressure levels in hypertension. Non-adherence contributes to poor blood pressure control, which increases the likelihood of stroke, myocardial infarction, heart failure, and renal disease [9,32]. Studies suggest that even modest improvements in medication adherence result in a meaningful reduction in systolic blood pressure and a corresponding decrease in cardiovascular risk [33]. Patients who adhere to their treatment regimens also report better health-related quality of life (HRQoL), as they are less likely to experience the debilitating effects of uncontrolled blood pressure [34].

In cardiovascular diseases, particularly heart failure and coronary artery disease, adherence to medications such as beta-blockers, angiotensin-converting enzyme inhibitors, and statins is crucial for reducing morbidity and mortality. Non-adherence in these conditions leads to worse clinical outcomes, including higher rates of hospital readmissions and cardiovascular mortality [35,36]. Evidence suggests that even small improvements in adherence can significantly reduce the risk of atherosclerosis progression, leading to fewer instances of heart attacks and strokes, and improve long-term survival rates in patients with cardiovascular diseases.

These findings suggest that improving adherence to therapy has a profound impact on the clinical outcomes of chronic diseases, leading to better disease control, fewer complications, and a reduced need for emergency interventions.

## 4. Adherence and Therapy Costs

The relationship between medication adherence and the associated healthcare costs is well-established in the medical and economic literature. Non-adherence to therapy leads to substantial direct and indirect economic costs. Direct costs refer to the financial outlays associated with increased use of healthcare services due to poor disease management. These costs include hospitalizations, emergency room visits, additional diagnostic testing, and more intensive treatments being required to manage complications that arise from suboptimal adherence [1].

Indirect costs, although less immediately visible, are equally significant. These costs encompass the loss of productivity due to increased absenteeism from work, long-term disability, and premature death. In chronic diseases such as diabetes, non-adherence not only results in more frequent hospital visits, but also leads to long-term complications such as blindness, kidney failure, and amputations, which severely impact an individual’s ability to work and to contribute economically [10]. Furthermore, the psychological and emotional toll on patients and their families, while difficult to quantify, constitutes another form of indirect cost. Patients who experience poor health outcomes due to non-adherence may face a diminished quality of life and psychological distress. For instance, the recurrence of health complications such as stroke or myocardial infarction due to non-adherence can severely limit a patient’s physical capabilities and independence, leading to a decreased quality of life [37]. Additionally, the emotional and mental stress associated with managing poorly controlled chronic conditions can contribute to increased rates of depression and anxiety, further complicating adherence behaviors.

Non-adherence to therapy places a significant financial burden on healthcare systems worldwide. In the United States alone, it is estimated that non-adherence costs the healthcare system between USD 100 billion and USD 300 billion annually due to avoidable hospitalizations, emergency room visits, and the treatment of preventable complications [38]. Similarly, in Europe, the economic burden of non-adherence is substantial, particularly in managing chronic diseases such as diabetes, cardiovascular disease, and chronic obstructive pulmonary disease. For example, in the United Kingdom, poor adherence to therapy is estimated to cost the National Health Service approximately GBP 500 million annually in additional hospitalizations and treatment expenses [39].

The financial burden is not limited to healthcare systems. Patients themselves also bear a significant portion of the costs. Non-adherence can lead to increased out-of-pocket expenses due to additional doctor visits, higher medication costs, and the need for more intensive care to manage complications. It has been described that the cost of non-adherence by the patient per year ranges from USD 949 to USD 52,341 [10]. This is especially concerning for patients from low-income backgrounds, for whom the cost of chronic disease management can already be a significant financial strain [40]. For example, a patient with poorly controlled diabetes may need more frequent specialist consultations, additional medication, and potential hospitalizations, all of which increase their personal healthcare expenditures.

It is important to indicate that there are sociodemographic differences, including gender and age differences, in cost-related medication non-adherence to prescribed medications. These differences mainly affect women and older people [41,42]. However, it should be noted that the impact of the availability of financial resources on cost-related medication non-adherence is related to the perceived affordability of drugs, access to healthcare, and overall patient satisfaction with healthcare services [43,44].

## 5. Adherence and Patient Quality of Life

Adherence to prescribed therapies is not only essential for achieving optimal clinical outcomes, but also plays a pivotal role in determining the quality of life of patients. The concept of health-related quality of life encompasses the physical, emotional, and social dimensions of well-being. Adherence to therapy has been shown to significantly improve HRQoL in patients with chronic conditions, primarily by enabling better disease control and reducing the occurrence of complications.

Studies have shown that patients who adhere to cardiovascular therapy experience fewer symptoms of heart failure, less fatigue, and better overall physical functioning compared to non-adherent patients [36]. The avoidance of disease exacerbation through adherence contributes to an enhanced perception of personal well-being, which translates into higher HRQoL scores. Similarly, in conditions like hypertension and chronic obstructive pulmonary disease, adherence to treatment is directly correlated with better HRQoL [45,46]. Non-adherent patients often suffer from a deterioration in physical function, a greater frequency of exacerbations, and higher rates of hospitalization, all of which negatively impact their daily living and HRQoL.

The physical benefits of adherence are evident in terms of improved disease control and reduced complications. This, in turn, allows patients to maintain their independence and engage in daily activities with fewer limitations. The physical benefits of adherence are not only limited to preventing adverse health events, but also extend to improving overall physical capacity, enabling patients to participate more fully in life.

Emotionally, adherence to therapy may reduce the psychological stress and anxiety associated with managing chronic diseases. Patients who adhere to their treatment regimens often experience a sense of control over their health, which mitigates feelings of helplessness or fear regarding the progression of their disease. On the contrary, non-adherence can lead to worsening symptoms and increased disease burden, which exacerbates anxiety and depression, particularly in patients with long-term conditions such as diabetes or heart disease. Poor emotional well-being, in turn, has been found to reduce adherence, creating a negative feedback loop that further undermines both mental health and clinical outcomes [47].

From a social perspective, adherence allows patients to maintain more normal social interactions and roles within their families and communities. When patients experience fewer symptoms or disease exacerbations due to adherence, they are more likely to participate in social activities, maintain employment, and fulfill family responsibilities. Non-adherence, on the other hand, often results in increased absenteeism from work, social isolation due to physical limitations, and a greater reliance on caregivers, all of which contribute to diminished social well-being [10]. Social support has also been identified as a critical factor in adherence, with patients who receive encouragement from family, friends, or healthcare providers being more likely to follow their prescribed therapies and thus improve their HRQoL.

## 6. Strategies to Improve Therapy Adherence: The Role of Healthcare Professionals and Technological Innovations

Several strategies have been developed to improve adherence to therapy, and many of these interventions have demonstrated positive effects on both clinical outcomes and HRQoL (Table 1).

One of the most effective strategies is patient education, which involves improving patients’ understanding of their condition and the importance of adhering to treatment. Educational interventions often include counseling sessions with healthcare providers, printed materials, or digital platforms that provide information on disease management [24,48]. By increasing patient knowledge and motivation, these programs enhance adherence and ultimately improve HRQoL by preventing complications and promoting a sense of control over the disease.

Technological innovations, such as mobile health applications and electronic medication monitoring systems, have also been shown to support adherence. These tools provide patients with reminders to take their medications, track adherence in real-time, and enable healthcare providers to intervene when necessary. Studies have shown that patients using such technologies report higher adherence rates and improved HRQoL, as they feel more connected to their care and are less likely to miss doses of medication [49]. Mobile applications, in particular, have been effective in chronic disease management by empowering patients to take an active role in monitoring their health, leading to better outcomes and higher satisfaction.

Technological innovations offer promising solutions for improving adherence, especially in the management of chronic diseases. Mobile applications (apps) are among the most widely used tools for supporting adherence. These apps often include features such as medication reminders, health tracking, and educational content. Studies have demonstrated that the use of mobile health apps improves adherence by helping patients to maintain a consistent medication schedule and providing them with real-time feedback on their progress [50]. For example, diabetes management apps that track blood sugar levels and medication intake can lead to better glycemic control and improved health outcomes.

In addition to mobile apps, electronic medication monitoring devices have emerged as a valuable tool in promoting adherence. These devices track when patients take their medication and provide healthcare providers with adherence data, enabling them to intervene when necessary. Electronic pillboxes that alert patients when it is time to take their medication have been shown to improve adherence, particularly in elderly populations who may struggle with complex regimens [51]. These devices reduce the risk of missed doses and help patients to stay on track with their treatment plans.

Telemedicine has also become a powerful tool in enhancing adherence, particularly in remote or underserved areas. Through telemedicine platforms, healthcare providers can conduct virtual consultations, monitor patient adherence, and provide real-time advice. Telemedicine is particularly beneficial for patients with chronic conditions that require continuous monitoring, such as hypertension or heart failure. By providing regular contact with healthcare providers, telemedicine can help to address adherence challenges early and prevent the escalation of health issues [52].

Another important strategy involves simplifying treatment regimens. Complex regimens with multiple daily doses are associated with lower adherence rates, as patients find it difficult to remember or manage the intake of multiple medications [53]. By simplifying the regimen, for instance, by prescribing once-daily medications or combination therapies, healthcare providers can significantly enhance adherence and improve patients’ HRQoL. Patients who follow simpler regimens report higher satisfaction and less burden from their treatments, which translates into better disease control and fewer disruptions to their daily lives.

Improving adherence to therapy is a multifaceted challenge that requires the coordinated efforts of healthcare professionals and the strategic application of technological innovations. Healthcare professionals are at the forefront of efforts to improve adherence, with physicians, pharmacists, and nurses playing vital roles in patient education and ongoing support. The physician’s role begins at the point of diagnosis and treatment planning, where clear communication is essential. Patients need to understand not only the benefits of adhering to their prescribed regimens, but also the potential consequences of non-adherence. Physicians who engage in shared decision making, where treatment options are discussed collaboratively with patients, tend to foster better adherence by making patients feel more involved in their care [54]. Additionally, simplifying treatment regimens and tailoring them to individual patient needs increases the likelihood of long-term adherence [53].

Pharmacists also play a critical role by providing medication counseling and ensuring that patients understand how to take their medications correctly. Pharmacist-led interventions, such as medication therapy management services, have been shown to significantly improve adherence, particularly in patients with chronic conditions like diabetes, hypertension, and asthma [55]. Preparing pill boxes is another tool that a pharmacist can use to support adherence [56]. By addressing patient concerns about side effects, dosage schedules, and potential drug interactions, pharmacists can help to alleviate barriers to adherence.

Nurses and other healthcare professionals contribute by providing ongoing support through regular follow-ups, monitoring patient progress, and identifying early signs of non-adherence. Nurses are particularly effective in chronic disease management programs, where frequent contact with patients helps to reinforce adherence behaviors and ensure that any obstacles to adherence are quickly addressed [57]. The role of healthcare professionals in fostering adherence is indispensable, as their support helps to build trust and engagement, which are key determinants of adherence.

Intervention programs that focus on improving adherence have the potential to reduce healthcare costs significantly. One of the most effective approaches involves the use of multidisciplinary teams that provide comprehensive patient support. These teams, often composed of physicians, pharmacists, nurses, and dietitians, work together to develop personalized care plans that address both clinical and adherence-related challenges [58]. Such programs have been particularly successful in reducing hospital readmissions and emergency room visits in patients with chronic conditions like heart failure and diabetes, resulting in substantial cost savings.

Another important intervention strategy involves financial incentives that reduce the cost burden on patients. High out-of-pocket costs for medications and treatments are a common barrier to adherence, particularly in low-income populations. Programs that reduce or eliminate co-pays for essential medications have been shown to improve adherence and lower overall healthcare costs by preventing costly complications and hospitalizations [59]. In addition, value-based insurance design programs, which reduce patient cost-sharing for high-value medications, have demonstrated improvements in adherence, particularly in patients with chronic diseases such as diabetes and hypertension [60].

Pharmacist-led interventions, such as medication synchronization programs, have also proven effective in improving adherence and reducing costs. Medication synchronization ensures that all of a patient’s medications are filled at the same time each month, reducing the complexity of managing multiple prescriptions and improving adherence [61]. Studies show that patients enrolled in synchronization programs are more likely to adhere to their medications and experience fewer hospitalizations and emergency room visits, leading to overall cost savings.

## 7. Conclusions

Adherence to therapy is fundamental to achieving successful clinical outcomes, reducing healthcare costs, and enhancing the quality of life for patients, particularly those with chronic conditions. This paper highlights the significant role of adherence not only in treatment efficacy, but also in strengthening healthcare systems and improving patient well-being. Beyond clinical outcomes, adherence reflects the trust, communication, and shared decision making between patients and healthcare providers, underscoring the need for a holistic, patient-centered approach.

However, barriers such as socioeconomic disparities, complex treatment regimens, and psychological challenges persist. Addressing these requires personalized strategies that cater to individual patient needs and equitable access to care. Future research should focus on the long-term effectiveness of digital health tools and innovative interventions to improve adherence across diverse populations.

## 8. Future Directions

Taking into account the literature review, in order to improve adherence to therapeutic recommendations, it is necessary to use various strategies simultaneously, including both an individual approach to the patient and a systemic approach. Considering the numerous factors influencing patients’ adherence to therapy, a personalized approach is extremely important, including a personalized selection of therapy, taking into account the patient’s psychological aspects, lifestyle, or financial possibilities. In addition, a multidisciplinary approach, including cooperation between physicians, pharmacists, and nurses in patient care, can further strengthen therapy adherence. In parallel, healthcare systems should develop educational programs covering the importance of adherence, which should be addressed to medical personnel (taking into account the need for a personalized approach to the patient), as well as to patients themselves.

Another direction of changes is the use of advanced technologies, such as mobile applications or monitoring devices, which enable constant support for patients in adhering to therapy. The development of digital tools that integrate medication reminders, real-time progress tracking, and access to educational content may be a breakthrough in increasing adherence. These technologies should be adapted to diverse patient groups, including the elderly, who may encounter barriers in using modern devices.

Such initiatives have the potential not only to enhance therapeutic outcomes and elevate patients’ quality of life, but also to significantly reduce healthcare expenditures.

## Figures and Tables

**Table 1:** Strategies to improve therapy adherence.

Strategy	Description	Benefits
Patient Education	Counseling and printed/digital materials to improve understanding of therapy.	Increased knowledge, adherence, and HRQoL.
Digital Health Tools	Mobile apps and electronic devices for reminders and tracking.	Enhanced engagement and better adherence.
Telemedicine	Virtual consultations and monitoring.	Early intervention and improved outcomes.
Simplified Regimens	Reducing complexity by prescribing once-daily medications.	Higher adherence and fewer complications.
Healthcare Professionals’ Involvement	Active involvement in fostering patient trust, providing continuous support, and encouraging adherence.	Improved patient–provider relationship, better adherence, and enhanced outcomes.
Intervention programs	Structured programs using multidisciplinary teams, financial incentives, and pharmacist-led interventions to improve adherence.	Reduced hospital readmissions, fewer emergency visits, enhanced adherence, and cost savings.
